# A community-based perspective

**DOI:** 10.4103/0019-5545.43055

**Published:** 2005

**Authors:** R. Parthasarathy

**Affiliations:** Professor and Head

Schizophrenia is a severe mental disorder affecting at least 1% of the population in developing countries. Experiences of schizophrenia often differ depending on one's socio-economic and cultural milieu. It has also been found that with appropriate treatment, most people can recover from schizophrenia and return to a normal life. Even those with persistent long-term schizophrenia can learn to manage their symptoms and live a productive life. In most societies, the disease carries a substantial stigma. Patients are often blamed for bringing on their illnesses and others may see them as victims. The illness may be attributed to fate, religious and moral transgression or witchcraft. Such stigma may keep families from acknowledging that a family member has schizophrenia.^3^ Some families may hide or overprotect a member with schizophrenia—keeping the person from receiving potentially effective care or they may reject the person. When magnified from individuals to a whole society, such attitudes lead to underfunding of the mental health services and inadequate care. In many villages, towns and cities, the mentally ill are chained, caged or hospitalized in filthy, brutal institutions. Schizophrenia almost always alters a person's life dramatically. People with schizophrenia experience disturbing symptoms that can make it difficult for them to hold a job, go to school, relate to others, or cope with the ordinary demands of life. Some individuals require hospitalization because they are unable to care for themselves or because they are at risk of committing suicide. They also suffer from other diseases and physiological ailments such as cardiovascular diseases at rates that exceed those of the average population. If left unrecognized and/or untreated, late-life schizophrenia may severely impair or even be fatal for those it afflicts.^4^

The costs differ on the basis of the type of person who has the illness—the head of the family, housewife, working woman, student; type of occupation; educational status; rural, urban or tribal background; or expectations and aspirations. In western settings, the economic cost alone is estimated. Even estimating the economic cost of schizophrenia is complex, because there are direct costs (hospital costs, health care provider fees and actual medical expenditure), indirect costs (cost of individuals and society due to reduced or lost productivity of family members) and support costs (time lost to care of family members with schizophrenia).

The debilitating nature of schizophrenia brings enormous family and societal costs that often go unnoticed, because they are not reported to mental health professionals or agencies. A considerable number commit suicide or become involved in violent activities that are harmful to themselves or to others. Hence, a variety of factors must be considered in developing assessment procedures to evaluate the cost of schizophrenia. Efforts to assess the cost of schizophrenia must also take into account the fact that its cost is not merely monetary, or only the cost of treatment. The overall cost of schizophrenia includes social and psychological costs experienced by patients and family members.^5^ For example, let us see the following situations:

*Housewife with schizophrenia:* One or two persons assist her or substitute for her, she tends to neglect household responsibilities, abandon the children, which affects their studies. It is also possible that her husband may remarry.

*Head of the family with schizophrenia:* The total income is lost, there are possibilities of irregular treatment and follow up, relapses and readmissions, aggressive and assaultative behaviour, decline in the quality of life. The patient is not bothered about family responsibilities, and children and other dependants are left in the lurch. The indirect excess cost due to unemployment is the largest component of the overall excess annual costs due to schizophrenia.

*Adolescents with schizophrenia:* There is interruption in or discontinuation of studies, future prospects are affected, this has a psychological impact on the parents or siblings, and growing stigma has to be dealt with.

*Marriageable girls with schizophrenia:* Their prospects of marriage are affected, persistent stigma affects other family members, these girls often face isolation and rejection and allied aspects.

In India, the majority of patients with schizophrenia are not under any treatment. The consequences of non-treatment are devastating:^6^

- *Homelessness:* People with untreated schizophrenia become homeless, the quality of life of these individuals is abysmal. Many are victimized regularly, they eat from garbage lying on the roadside. They lead their life in the streets.
- *Incarceration:* People with untreated schizophrenia comprise a significant percentage of the prison inmate population. These individuals are often incarcerated with charges of misdemeanour, but sometimes with charges of felony caused by their psychotic thinking. It has been found that people with untreated psychiatric illnesses spend twice as much time in jail than individuals who are not ill, and are more likely to commit suicide.
- *Episodes of violence:* Several murders are committed each year by people with untreated schizophrenia. Spouses are killed by their spouses, children killed by parents, parents killed by children and siblings killed by siblings. In such cases, untreated schizophrenia plays a vital role.
- *Victimization:* Most crimes against individuals with severe psychiatric disorders are not reported; in those instances where they are reported, officials often ignore them; it is not rare for them to be victims of rape and murder.
- *Suicide:* Suicide is the number one cause of premature death among people with schizophrenia. The extreme depression and psychosis that can result due to lack of treatment are the usual causes of death in these sad cases.
- *Clinical outcomes more severe—recovery uncertain:* The longer the individual with schizophrenia remains untreated, the more uncertain their prospects for long-term recovery.
- *Relapse:* Relapses, readmissions due to various factors drain the meagre resources of the government, family and community. The monthly relapse rates are estimated to be 3.5% per month for patients on maintenance neuroleptics and 11.0% per month for patients who have discontinued their medication. Post discharge non-compliance rates in community settings are estimated to be 7.6% per month.^7^

Considering all these facts many of the aspects of schizophrenia remain immeasurable.^8^ The following list indicates the nature of the immeasurable costs.

Invaluable time spent; insignificant life led
Monitoring of day-to-day activities highly taxing; memories bitter
Mood instability and reaction of family members, multiple problems
Expectations collapse; exaggerated blame
Ambitions unfulfilled; aggressive and assaultive mood
Support weaned; skills lost; status diminishes
Uselessness felt; unusual bizarre behaviour embarrassing
Reputation declines—resilience challenged
Ambiguity about the future; achievement, affection and acceptance unattained
Boredom mounts; blackout of potentials
Lifelessness—feelings of loneliness
Esteem loss; elevated dilemma; estrangement

Comfort loss—callousness troubles
Overall stress; oscillating lifestyle
Stigmatizing struggles; sarcasm
Traumatic events; tranquillity torn

By analysing all such factors related to the cost of schizophrenia, one can infer that the economic cost of schizophrenia is just the tip of the iceberg. Other aspects which are ‘hidden’ and ‘invisible’ are, in fact, more expensive than what is visible and obvious. Hence, mental health professionals need to think of understanding the cost of illness from different angles so that comprehensive strategies dealing with psychological, social, cultural and economic issues can be formulated at the micro- and macro levels. Some of them could be as follows:

1. All efforts to prevent relapses: Interdisciplinary approaches, teamwork, active family involvement and utilization of community resources, influencing policy issues, etc.
2. Provision of community-based care: Treatment facilities should be made available in nearby areas—PHCs, PHUs, general hospitals, better referral system and linkages to community mental health institutes.
3. Use of new antipsychotic medication: Innovative drugs called ‘atypical antipsychotics’ including clozapine, risperidone and olanzapine should be used for the treatment of both the positive and the negative symptoms of schizophrenia.
4. Psychoeducation for family members:^9^ Multiple psychoeducational family group interventions are effective in handling the negative symptoms which are associated with relapse, poor social and occupational functioning, cognitive impairment and lower subjective quality of life.
5. Community awareness about schizophrenia: Concerted efforts should be made by effectively using the mass media to create positive awareness among policy-makers, administrators, educationalists, welfare personnel, voluntary agencies and others interested in the welfare of the mentally ill.
6. Comprehensive rehabilitation for patients: Intersectoral collaboration, cooperation and support are required from governmental and non-governmental agencies for the purpose of creating rehabilitation services such as day-care programmes, sheltered workshops, halfway homes, etc.
7. Appropriate job opportunities for the mentally restored person: Active implementation of legislative measures connected with disabilities so that benefits reach the needy in the community.
8. Governmental support to voluntary agencies involved in care and rehabilitation activities: Certain financial and other support can be extended to voluntary agencies to run programmes for patients and families.
9. Adequate treatment facilities in rural and tribal areas: Equitable distribution of services so that even in the remotest villages as well as tribal areas appropriate psychiatric services are made available.
10. Implementation of National Mental Health Programmes in letter and spirit: The tenets of National Mental Health programmes are translated into practice throughout the nation by means of District Mental Health Programmes, intersectoral cooperation and coordination, etc.

Early diagnosis and better treatment adherence, family centred and community-based care programmes, incorporating clinical case management and improving treatment and rehabilitation are expected to lower the overall costs while improving the quality of life and social functioning of patients.
